# The Ace of Spades: Reverse Takotsubo Cardiomyopathy in the Context of Angiographic Embolization of Recurrent Metastatic Serotonin-Positive Neuroendocrine Tumour of the Pancreas

**DOI:** 10.1155/2013/793193

**Published:** 2013-02-07

**Authors:** Ian A. Mazzetti, Michael J. Marcaccio, Odette Boutross-Tadross, Omid Salehian, Catherine Demers

**Affiliations:** ^1^McMaster University, 1280 Main Street West, Hamilton, ON, Canada L8S 4K1; ^2^Department of Surgery, McMaster University, 1280 Main Street West, Hamilton, ON, Canada L8S 4K1; ^3^Department of Pathology and Molecular Medicine, McMaster University, 1280 Main Street West, Hamilton, ON, Canada L8S 4K1; ^4^Division of Cardiology, McMaster University, 1280 Main Street West, Hamilton, ON, Canada L8S 4K1

## Abstract

A 62-year-old woman undergoing embolization of recurrent neuroendocrine tumor, positive for serotonin, developed chest pain and bradycardia with lateral ST-segment depression. Cardiac biomarkers were elevated, and echocardiography revealed akinesis of all basal segments with a normally contracting apex. The absence of flow-limiting coronary disease on angiography confirmed the presence of reverse Takotsubo cardiomyopathy. After optimal medical therapy for six weeks, left ventricular function returned to normal. Takotsubo cardiomyopathy has been described across a wide variety of hyperadrenergic states; the description of the reverse-type Takotsubo cardiomyopathy in the setting of embolization of recurrent neuroendocrine with serotonergic positivity tumour is novel.

## 1. 60-Word Summary

A 62-year-old woman undergoing embolization of recurrent serotonergic neuroendocrine tumor developed chest pain with lateral ST depression. Cardiac biomarkers were elevated, and echocardiography revealed akinesis of all basal segments with a normally contracting apex. The absence of flow-limiting coronary disease on angiography confirmed the presence of reverse Takotsubo cardiomyopathy. The description of reverse-type Takotsubo cardiomyopathy in this setting is novel.

## 2. Case Description

**Figure 1:**
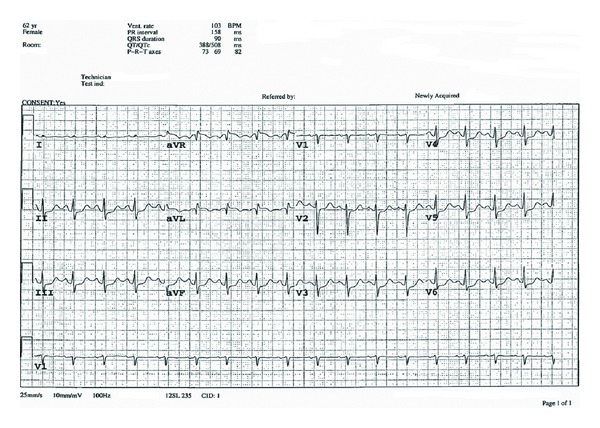
Initial 12-lead electrocardiogram showing diffuse ST segment depression (predominantly involving the lateral precordial and inferior leads) with minor ST elevation in lead aVL.

**Figure 2:**
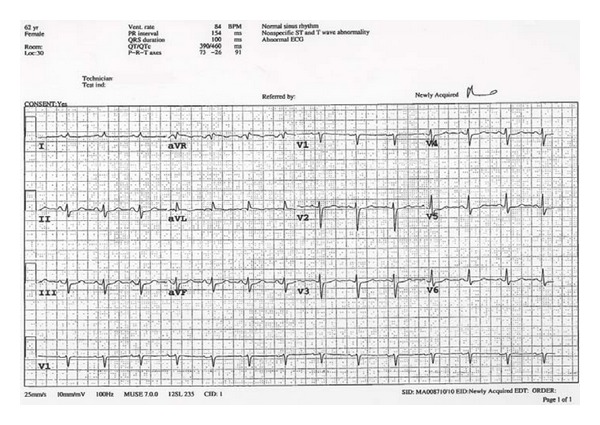
Subsequent 12-lead electrocardiogram performed after 24 hours shows resolution of ST segment changes.

A 62-year-old woman undergoing angiographic bland embolization of the right hepatic artery by interventional radiology service for liver metastases from a previously resected neuroendocrine tumor of the pancreas, which stained positive for serotonin, developed severe chest and epigastric pain radiating to the back during the procedure. She had been pretreated with octreotide. Initial 12-lead electrocardiogram (ECG) revealed sinus rhythm and lateral and inferior ST-segment depression as well as ST elevation in lead aVL (Figure 1), while initial cardiac biomarkers were elevated (troponin T 0.85 *μ*g/L, peak 1.08 *μ*g/L; CK 652 U/L). The patient had no history of cardiac disease or cardiac risk factors, while significant medical history included hypothyroidism, psoriatic arthritis, and pancreatic neuroendocrine carcinoma resected a year earlier. After CT scan ruled out aortic dissection (a rare complication of abdominal angiography), she was transferred to the Coronary Care Unit and treated for an acute coronary syndrome. As the embolization procedure was performed by the interventional radiologist in a hospital where there is no cardiac catheterization available, the patient was not immediately referred for a coronary angiogram. A subsequent ECG performed after 24 hours (Figure 2) revealed the resolution of ST segment changes. 

**Figure 3:**
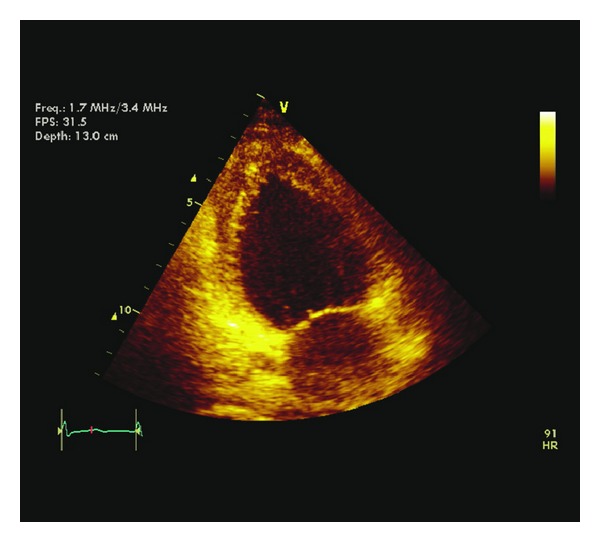
Apical 2-chamber view during systole showing basal hypokinesis and normal apex, the “ace of spades” appearance of left ventricular contractile function.

**Figure 4:**
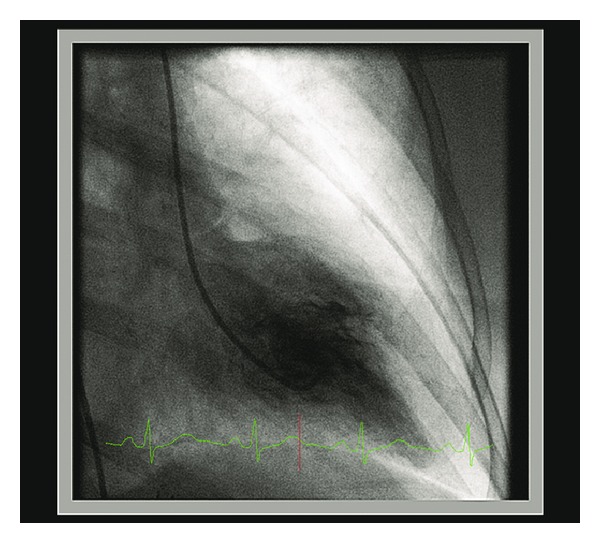
Left ventricular angiogram showing the “ace of spades” appearance of left ventricular contractile function, with basal hypokinesis and normal apex, consistent with reverse Takotsubo cardiomyopathy.

 Echocardiography was carried out the next day, revealing a normally contracting left ventricular (LV) apex, but akinesis of all basal segments and hypokinesis of the mid-ventricular segments with LV ejection fraction estimated at 40% with no significant valvular dysfunction (Figure 3, Supplementary Movie I in Supplementary Material available online at http://dx.doi.org/10.1155/2013/793193). Coronary angiography revealed normal coronary arteries in keeping with a nonischemic cardiomyopathy, while ventriculography confirmed the echocardiographic findings, showing severe hypokinesis of the antero- and inferobasal segments with normal contractile function of the more distal segments (Figure 4, Supplementary Movie II). These findings were consistent with reverse-type Takotsubo cardiomyopathy. During her stay in the coronary care unit she had stable hemodynamics and at no point required inotropic or vasopressor support. She was treated medically with combination of ACE inhibitor, beta blocker, and antiplatelet therapies, and repeat echocardiography performed six weeks later showed significant improvement in LV function with ejection fraction in the low normal range at 50%, with very mild residual hypokinesis of the basal segments alone (Supplementary Movie III) and no significant valvular dysfunction, confirming the diagnosis of reverse-type Takotsubo cardiomyopathy.

## 3. Discussion

Takotsubo cardiomyopathy, known variably as apical ballooning syndrome, stress-induced cardiomyopathy, or “broken heart syndrome,” was first described in 1991 and is characterized by severe, reversible LV dysfunction not attributable to flow-limiting coronary artery disease [1]. The precise incidence is unknown, with varied presentation and diagnostic criteria, but several studies estimate that 1%-2% of patients diagnosed with acute coronary syndrome actually have apical ballooning syndrome [2]. It would seem the incidence is on the rise due to the increasing use of early angiography and ventriculography in the setting of acute coronary syndrome. This unique cardiomyopathy usually occurs in postmenopausal women, with about 90% of all cases in the literature being described in women [2]. Four types have been described: classic type, with apical ballooning; reverse type, with hyperdynamic apex and basal akinesis; mid-ventricular type, which spares the base and apex; and localized wall motion abnormality of the anterior wall [3]. Our case is consistent with the reverse type, with severe wall motion abnormalities in the basal segments and normally contracting apical segments seen on echocardiography and ventriculography (Supplementary Movies II and III).

The reverse type is rarely described in the literature [3, 4], having been first described in 2006. In a recent publication which evaluated the prevalence of stress cardiomyopathy, only 1 percent of the population presented with the reverse or basal form of regional ballooning [5].

The etiology of Takotsubo cardiomyopathy remains elusive. Sympathetic activity and the role of catecholamines leading to myocardial stunning and toxicity, as well as coronary artery vasospasm, have been implicated, as has estrogen withdrawal [5], but the evidence for any one hypothesis is not compelling [5]. Density of sympathetic nerves, ß-receptors, and regional differences in adrenergic sensitivity may have a role in the wall motion abnormalities seen in the typical and reverse patterns [3]. In a case series of 19 patients with LV dysfunction (mean ejection fraction 20%) after sudden emotional stress, endomyocardial biopsy showed mononuclear infiltrates and contraction-band necrosis, while plasma catecholamine levels were higher among patients with stress-induced cardiomyopathy than among those with Killip class III myocardial infarction [5], suggesting a role for myocardial toxicity, stunning, and catecholamines. Animal studies suggest diminished sympathetic innervation of the left ventricle moving from base to apex, explaining the typical Takotsubo pattern, but a pathophysiologic rationale for the reverse type is lacking [1]. The basis of the preponderance of women towards Takotsubo cardiomyopathy is unknown. Sex hormones have important influences on the sympathetic neurohormonal axis, but sex-related differences on coronary vasoreactivity and catecholamine responsiveness remain poorly characterized [6]. Release of serotonin may be associated with an unopposed vasoconstricting effect leading to abnormal wall motion abnormalities [7].

Takotsubo cardiomyopathy has been described across a wide spectrum of precipitating causes including severe emotional distress or acute medical illness, physical distress (e.g., judo competitions), occult pheochromocytoma, amphetamine use, and the stress and pain associated with elective surgery [5].

**Figure 5:**
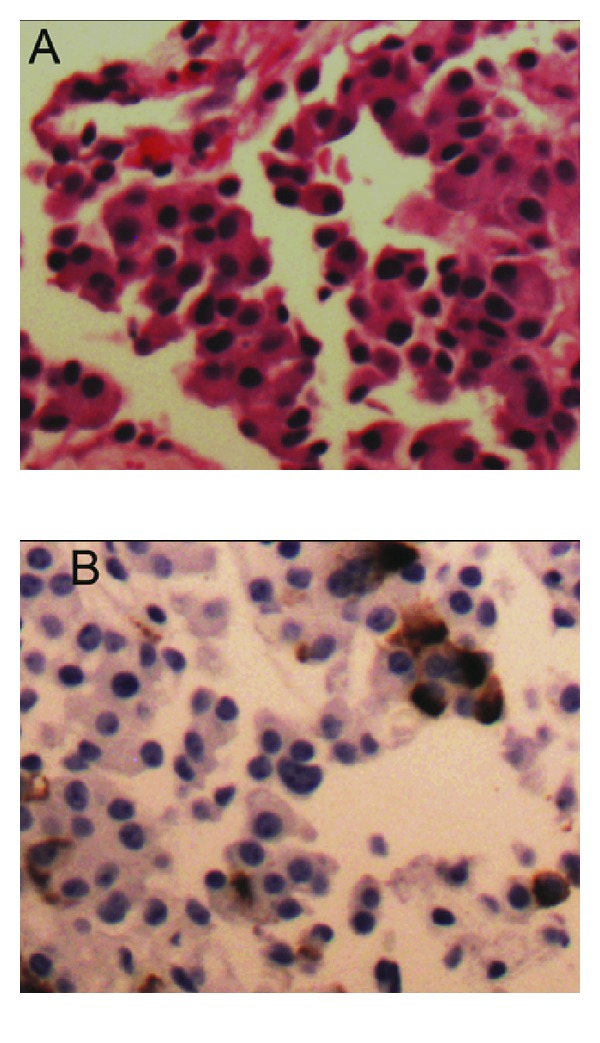
Core biopsy of liver, showing metastatic neuroendocrine carcinoma (×10 obj). (A) Hematoxylin/Eosin. (B) Biopsy showing focal positivity for serotonin.

In this case, it is difficult to separate the hyperadrenergic state inherent to the embolization procedure and the role of vasoactive hormones secreted by our patient's recurrent neuroendocrine tumour as the precipitant of the reverse Takotsubo cardiomyopathy. Her postmenopausal status and the stressful situation of an invasive procedure for recurrent metastatic tumour were clear risk factors. Immunohistochemistry of the primary tumour a year prior revealed focal positivity for serotonin (Figure 5), as well as insulin, glucagon, and pancreatic polypeptide. Case reports in the literature exist that describe an association between serotonergic states (e.g., use of duloxetine and the serotonin syndrome) and stress cardiomyopathy, of both the classic and reverse Takotsubo pattern [8]. However, in the absence of elevated 24-hour hydroxyindoleacetic acid levels (normal in our patient at two-year followup for her neuroendocrine tumour), any attribution of mechanism is speculative. 

We have described a unique case of reverse Takotsubo cardiomyopathy in the context of angiographic embolization of a recurrent neuroendocrine tumour. Knowledge of this association may be useful in the perioperative, neuroendocrine oncology, and interventional radiology spheres. 

## Supplementary Material

Movie I. Echocardiogram (apical 4-chamber view) showing dilated left ventricle with moderate systolic dysfunction (left ventricular ejection fraction 40%), with segmental wall motion abnormalities: hypokinetic mid-anterior wall with akinetic interventricular septum, basal inferolateral and anterior walls. The apical segment contracts normally.
Movie II. Left ventricular angiogram in the RAO projection, showing severe hypokinesis of the basal segments of the anterior and inferior walls, with normal contractile function of the more distal segments and apex.
Movie III. Repeat echocardiogram (apical 4-chamber view) showing normal left ventricular cavity size with very mild hypokinesis of the basal inferior and inferoseptal wall with a low normal systolic function and mild degree of diastolic dysfunction.
